# Self-Assembled Benznidazole-Loaded Cationic Nanoparticles Containing Cholesterol/Sialic Acid: Physicochemical Properties, In Vitro Drug Release and In Vitro Anticancer Efficacy

**DOI:** 10.3390/ijms20092350

**Published:** 2019-05-11

**Authors:** Alaine Maria dos Santos-Silva, Lilia Basílio de Caland, Ednaldo Gomes do Nascimento, Ana Luiza C. de S.L. Oliveira, Raimundo F. de Araújo-Júnior, Alianda Maira Cornélio, Matheus F. Fernandes-Pedrosa, Arnóbio Antônio da Silva-Júnior

**Affiliations:** 1 Laboratory of Pharmaceutical Technology and Biotechnology, Department of Pharmacy, Federal University of Rio Grande do Norte, UFRN, Gal. Gustavo Cordeiro de Farias, Petrópolis, Natal 59.072-570, Brazil; alaine.maria@hotmail.com (A.M.d.S.-S.); liliabasiliocaland@gmail.com (L.B.d.C.); ednaldogn40@gmail.com (E.G.d.N.); mffpedrosa@gmail.com (M.F.F.-P.); 2 Department of Morphology, Federal University of Rio Grande do Norte, UFRN, Natal 59078-970, Brazil; analu.cs@hotmail.com (A.L.C.d.S.L.O.); araujojr@cb.ufrn.br (R.F.d.A.-J.); alianda@neuro.ufrn.br (A.M.C.)

**Keywords:** functionalization of nanoparticles, surface of nanoparticles, structural properties, cationic nanoparticles, biodegradable nanoparticles

## Abstract

Cationic polymeric nanoparticles (NPs) have the ability to overcome biological membranes, leading to improved efficacy of anticancer drugs. The modulation of the particle-cell interaction is desired to control this effect and avoid toxicity to normal cells. In this study, we explored the surface functionalization of cationic polymethylmethacrylate (PMMA) NPs with two natural compounds, sialic acid (SA) and cholesterol (Chol). The performance of benznidazole (BNZ) was assessed in vitro in the normal renal cell line (HEK-293) and three human cancer cell lines, as follows: human colorectal cancer (HT-29), human cervical carcinoma (HeLa), and human hepatocyte carcinoma (HepG2). The structural properties and feasibility of NPs were evaluated and the changes induced by SA and Chol were determined by using multiple analytical approaches. Small (<200 nm) spherical NPs, with a narrow size distribution and high drug-loading efficiency were prepared by using a simple and reproducible emulsification solvent evaporation method. The drug interactions in the different self-assembled NPs were assessed by using Fourier transform-infrared spectroscopy. All formulations exhibited a slow drug-release profile and physical stability for more than 6 weeks. Both SA and Chol changed the kinetic properties of NPs and the anticancer efficacy. The feasibility and potential of SA/Chol-functionalized NPs has been demonstrated in vitro in the HEK-293, HepG2, HeLa, and HT-29 cell lines as a promising system for the delivery of BNZ.

## 1. Introduction

Many anticancer drugs induce the generation of intracellular reactive oxygen species (ROS), which cause oxidative stress and cell damage [1]. The main use of the drug benznidazole (BNZ) is for the treatment of Chagas disease, an important neglected global parasitic disease caused mainly by the parasite *Trypanosoma cruzi* [2]. Mechanistically, the nitro group of BNZ is reduced in cells to an amino group, which produces ROS and subsequently induces cell apoptosis [3,4]; therefore, it may also be a potential anticancer drug. Recently, we demonstrated the improved biological efficacy of BNZ in human HT-29 colorectal cancer cells through the use of stable, biocompatible, and small-sized cationic nanoparticles (NPs) [5]. 

Poorly water-soluble drugs, such as BNZ, exhibit enhanced toxicity and decreased efficacy due to their limited ability to target the affected tissues. In addition, the oxidative stress induced in cells by chemotherapy is known to cause side effects in patients with cancer. Consequently, many studies have focused on the design of polymeric NPs as promising drug targeting systems [6,7]. The copolymer of N,N-dimethylaminoethyl methacrylate (DMAEMA) with methylmethacrylate (MMA) and butylmethacrylate (BuMA), (PDMAEMA-co-MMA/BMA, 2:1:1 mole ratio) is a well-used cationic polymer for this purpose. It has interesting properties, such as biocompatibility, low toxicity, bioadhesion, and an excellent adsorption capacity [8,9]. In addition to the physical stability, drug protection, and slow drug release, these particles have the ability to escape from the mononuclear phagocyte system (MPS) and overcome biological barriers [10,11]. 

The favorable electrostatic interaction of NPs with negatively charged phospholipids from biological membranes enhances drug uptake by endocytosis and improves anticancer drug efficacy [12]. Their escape from the degradative environment of the endosomes/lysosomes after internalization is also expected, owing to destabilization of membrane [13,14]. However, highly cationic NPs have been associated with some non-specific limitations or toxicities [11,15]. Strongly cationic NPs can be rapidly cleared from blood circulation [16]. Some previous studies have also reported the induction of toxicity by the generation of ROS and cell-membrane disruption [17]. 

Thus, tailoring the surface characteristics of cationic NPs can further decrease their cytotoxicity and improve their ability to enhance the specific uptake of the drug by cancer cells [18,19]. In addition, the physical instability of polymeric NPs can be mitigated by the modification of the NP surface with polymers, surface-active molecules, or natural compounds [19,20,21]. For this purpose, sialic acid (SA) and cholesterol (Chol) are interesting molecules for the surface functionalization of NPs. These compounds are endogenous substances involved in several physiological processes within cells [22,23,24,25]. The conjugation of SA and Chol to NPs is possible with reactive functional groups of the polymers, such as thiol, carboxyl, or amino groups, through covalent binding or electrostatic interaction [26,27].

In this study, the effects of surface modification of cationic NPs induced by SA and Chol were explored to improve cellular uptake of the chemotherapeutic agent benznidazole in three cancer cell lines and one normal cell line. The interactions among the involved compounds and physicochemical properties of the different formulations were monitored by using multiple analytical approaches. The enhancement of the anticancer efficacy of benznidazole-loaded cationic NPs conferred by SA or Chol is an interesting and valuable result, that has not been previously reported, and may result in practical gains in preformulation studies. 

## 2. Results

### 2.1. Preparation and Characterization of Surface-Modified Chol/SA NPs

The physicochemical properties of the different formulations of cationic NPs are shown in Table 1. The selected parameters for emulsification with the solvent evaporation technique successfully induced the formation of small (<150 nm) NPs with a narrow size distribution (PdI < 0.3). The zeta potential (+25 mV) indicated the cationic character of NPs. Analysis of the drug-loaded NPs (NP BNZ) revealed the high drug-loading efficiency (>85%) of the method. A 1:40 (SA/Chol):copolymer ratio was selected for further studies as it yielded the highest drug-loading efficiency. The following differences were observed between NP BNZ and the surface-modified formulations: A slight alteration in zeta potential, increased particle diameter, and considerably decreased encapsulation efficiency. 

**Figure 1:**
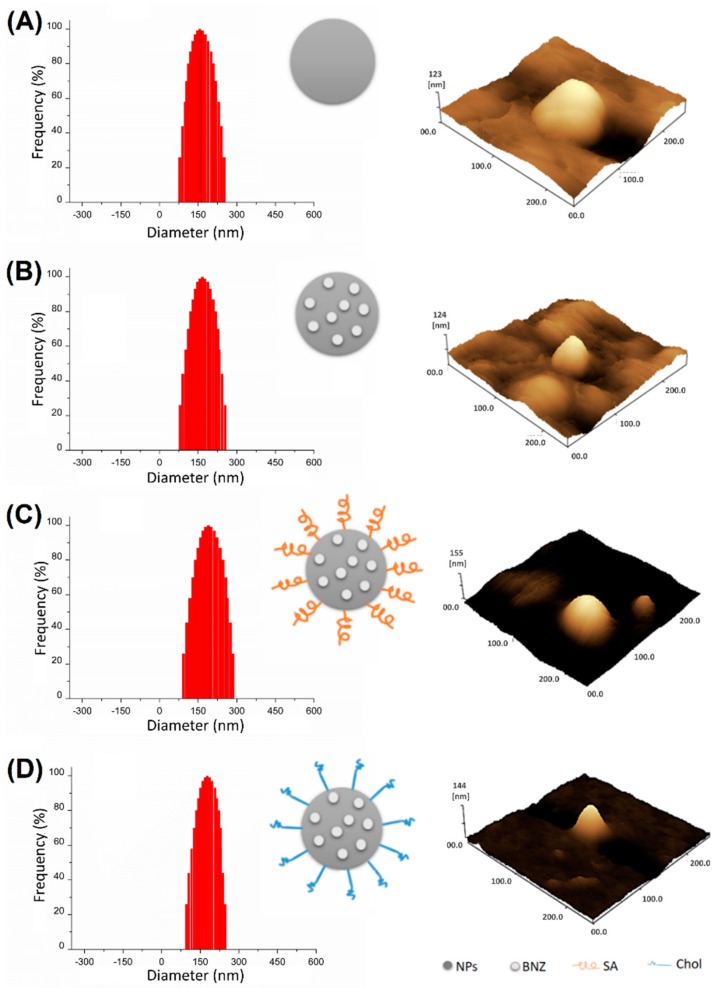
Particle size distribution (left) and 3D AFM images (right) of (**A**) NPs; (**B**) NP BNZ; (**C**) NP BNZ SA; and (**D**) NP BNZ Chol.

The particle size distribution and 3D AFM images of the formulations selected for further study are shown in Figure 1. The images show the formation of uniformly distributed sub-200 nm spherical NPs, with an apparently smooth surface. The comparison of drug-free NPs (Figure 1A) and drug-loaded NPs (Figure 1B) confirmed that drug-loading did not affect these parameters. Finally, drug-loaded NPs preserved these desired properties, even after functionalization with SA (Figure 1C) or Chol (Figure 1D).

**Figure 2:**
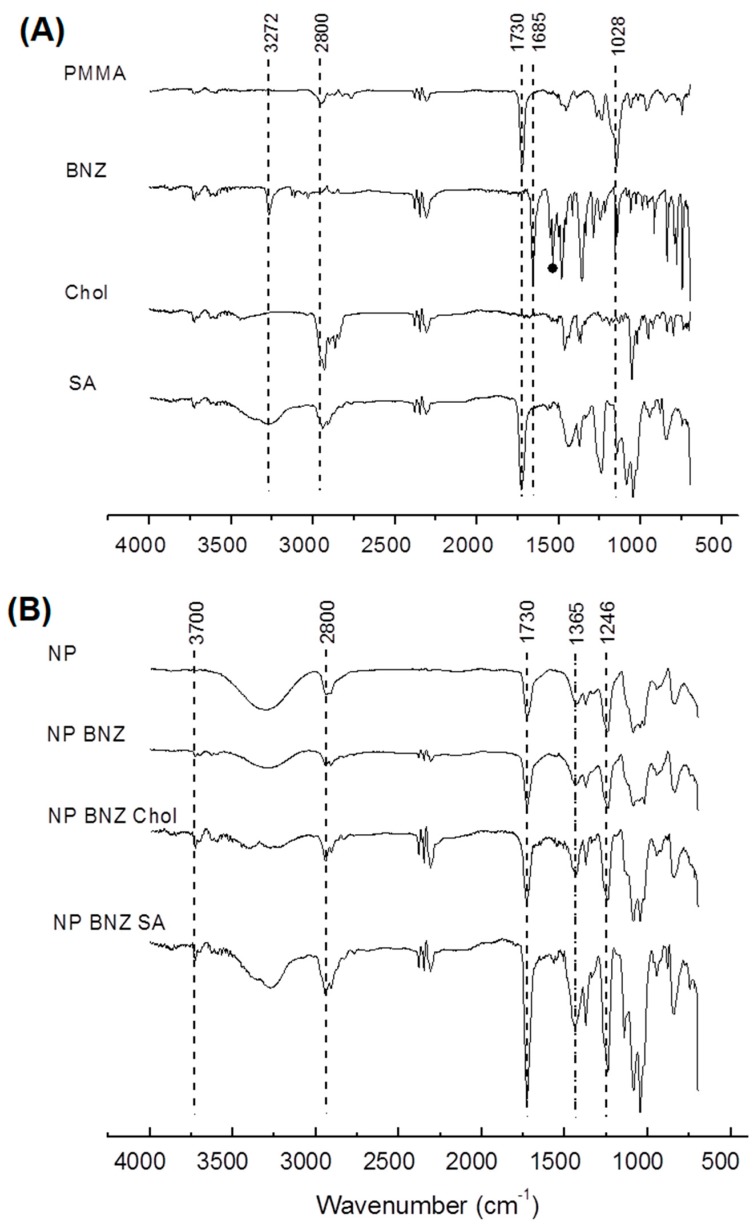
ATR-FTIR spectra of the following: (**A**) pure compounds (PMMA, BNZ, Chol, and SA), and NPs no drug (NPs); and (**B**) NPs containing BNZ (NP BNZ), nanoparticles, Chol-functionalized NPs containing BNZ (NP BNZ Chol), and SA-functionalized containing BNZ (NP BNZ SA).

The FTIR spectra of the pure compounds and the different formulations are shown in Figure 2A,B, respectively. PMMA displays a characteristic absorption band of an ester at approximately 1750 cm^−1^ and of N-C at 1246 cm^−1^. The spectrum of BNZ showed characteristic absorption bands at 3180 cm^−1^ for N-H axial deformation of secondary amides. The stretching of the NO_2_ group was identified in the range of 1685 cm^−1^, at 1530–1470 cm^−1^ and 1365–1318 cm^−1^. The angular C-H deformation in the plane and out of the plane of the bonds of aromatic compounds was identified in the range of 1246–1028 cm^−1^ [28]. Chol displayed the C-O stretching of the alcohol at approximately 1050 cm^−1^. The stretching O-H band from the carbonyl group of SA was identified at approximately 3300 cm^−1^, with the C-O stretching vibration in the range 1750–1730 cm^−1^. The secondary amide was at approximately 1630 cm^−1^. 

The comparison of PMMA (Figure 2A) with NPs (Figure 2B) mainly revealed the hypsochromic shift of the ester band to 1730 cm^−1^ and the N-C band to 1246 cm^−1^, due to different intermolecular interactions of the copolymer with the NPs. When the spectrum of NP was compared with NPBNZ (Figure 2B), overlapping of the PMMA bands was observed, which masked the BNZ bands. However, a slight enhancement of the marked bands was identified in the spectrum of NPBNZ. This enhancement was more clearly observed in the spectra of the functionalized NPs (NP BNZ SA and NP BNZ Chol).

### 2.2. Physicochemical Stability Study

**Figure 3:**
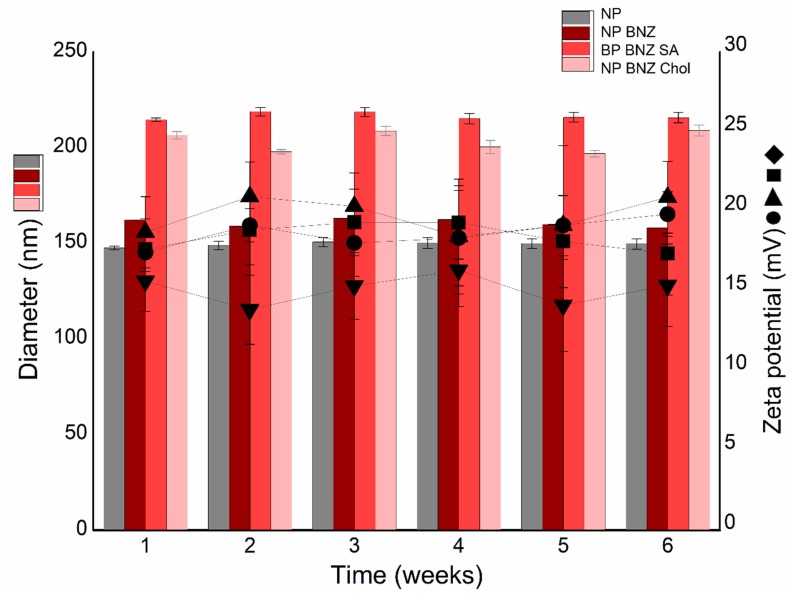
Mean diameter and zeta potential as a function of storage time for (▼) NP, (■) NP BNZ, (▲) NP BNZ SA, and (●) NP BNZ Chol. Note: The data are expressed as the mean ± standard deviation (SD) (*n* = 3).

The physicochemical stability of the different formulations is shown in Figure 3. The experimental data confirmed the excellent and extraordinary stability of NPs. The NP formulation remained stable (<150 nm) with a zeta potential of approximately +20 mV over the entire study period (6 weeks). The drug-loaded NPs (NP BNZ) and subsequent functionalization with SA (NP BNZ SA) or Chol (NP BNZ Chol) did not affect the particle size or zeta potential parameters. No significant changes (*p* > 0.05) were observed when the data for specific intervals were compared with the first week. 

### 2.3. In Vitro Drug Release Kinetics

**Figure 4:**
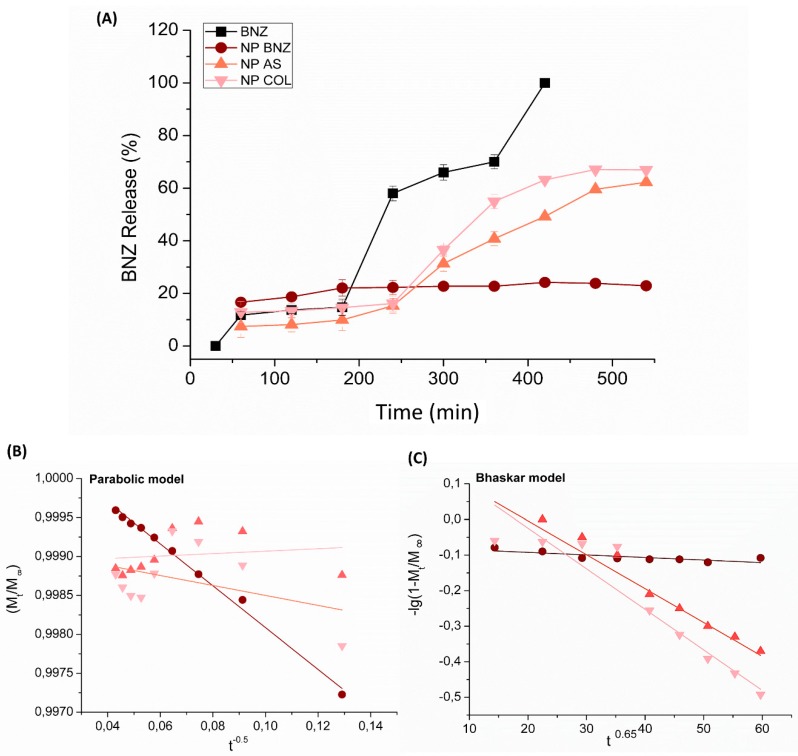
(**A**) Experimental in vitro drug release profile from different formulations. Respective mathematical modeling adjustment of data using (**B**) a parabolic model and (**C**) the Bhaskar model. Notes: The different samples can be identified as follows: (■) Solution of pure BNZ; (●) NP BNZ; (▲) NP BNZ SA; and (▼) NP BNZ Chol. The data are expressed as the mean ± standard deviation (SD) (*n* = 3).

Experimental data of the drug release from different formulations are presented in Figure 4. All tested formulations showed a slower release profile than the free drug (BNZ) (Figure 4A). In the first 120 min, a reduced burst effect (release of approximately 20%) was observed for NP BNZ, which was lower for functionalized NPs. However, the functionalization enhanced the drug release rate after 360 min. These differences were better evaluated by using four kinetic models, as follows: First-order; Bhaskar; modified-Freundlich; and a parabolic model. The fitting parameters are shown in Table 2. In the equations, Mt/M∞, t, and k correspond to the fraction of released drug, release time, and release rate constant, respectively. The drug release profile from non-functionalized NPs was a better fit to the parabolic model, with a linear correlation coefficient above 0.99 (Figure 4B).

For the functionalized NPs, the model that the best fit was Bhaskar (Figure 4C), with correlation coefficients of 0.93 and 0.94 for the NPs containing Chol and SA, respectively.

### 2.4. In Vitro Anticancer Efficacy

**Figure 5:**
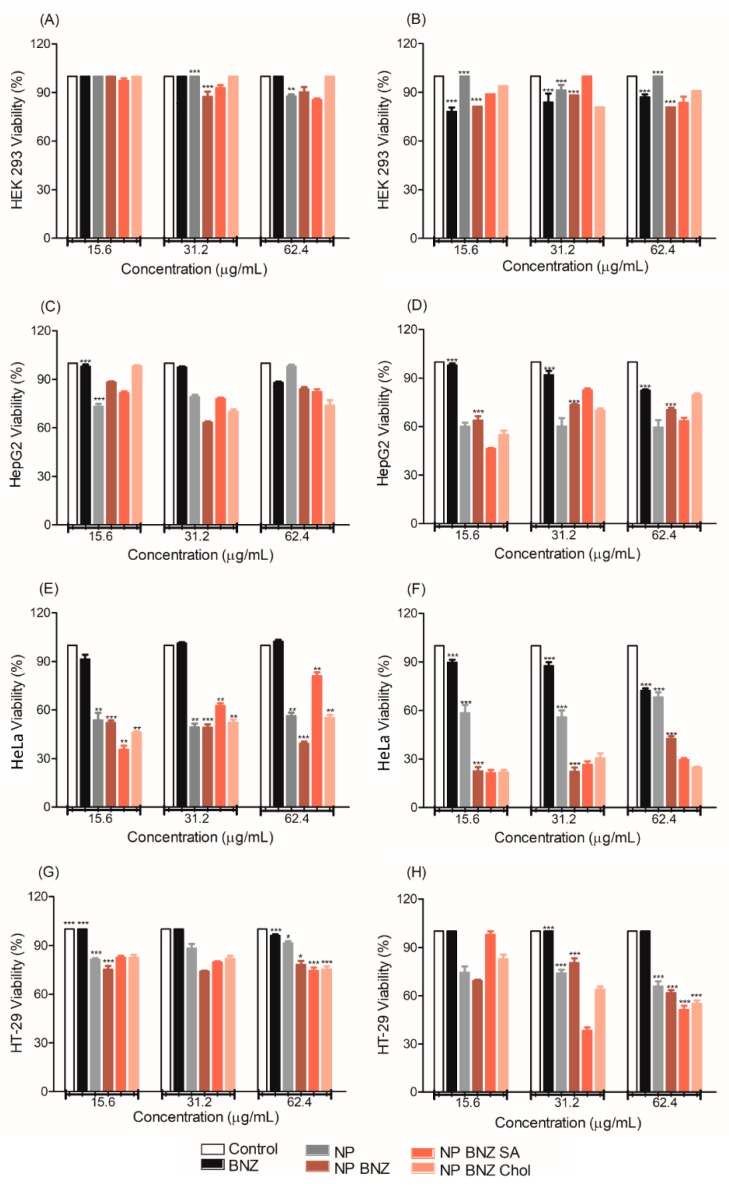
Cytotoxicity of the control (DMEM supplemented with 10% (*v*/*v*) fetal bovine serum), benznidazol (BNZ), drug-free NPs (NP), drug-loaded NPs (NP BNZ), SA-functionalized drug-loaded NPs (NP BNZ SA), and Chol-functionalized drug-loaded NP (NP BNZ Chol), as a function of test concentration, as determined by the MTT assay in four different cell lines after 24 h and 48 h of incubation. (**A**,**B**) Normal kidney HEK 293 cell line, (**C**,**D**) Human hepatocyte carcinoma (HepG2) cell line, (**E**,**F**) Human carcinoma cervical cancer (HeLa) cell line, and (**G**,**H**) human colorectal cancer (HT-29) cell line. Notes: The data are expressed as the mean ± standard deviation (SD) (*n* = 3), (*): *p* < 0.05, (**): *p* < 0.01, and (***): *p* < 0.001.

The viability of cells treated with different formulations, determined using the MTT assay, is shown in Figure 5. The treatment of normal kidney HEK 293 cells with all tested formulations resulted in no cytotoxicity at 24 h (Figure 5A) and 48 h (Figure 5B). The NPs appeared to increase the cytotoxicity of the drug to the human hepatocyte carcinoma (HepG2) cell line (Figure 5C,D). This effect was more clearly apparent in the human cervical carcinoma (HeLa) cell line (Figure 5E,F), and the treatment of the human colorectal cancer (HT-29) cell line (Figure 5G,H) reinforced this achievement. It was only possible to highlight the effect of functionalization on HeLa (Figure 5F) and HT-29 (Figure 5H) cells, as the NPs functionalized with SA (NP BNZ SA) and Chol (NP BNZ Chol) were more cytotoxic than NP BNZ.

## 3. Discussion

An initial screen of three different (SA/Chol) copolymer ratios revealed that the highest drug-loading efficiency was achieved for 1:40 *w*/*w* (data not shown). The data in Table 1 showed that both SA or Chol enhanced the particle size and decreased the drug-loading efficiency of BNZ NPs. The small changes in zeta potential corroborated with the perturbation on the particle surface induced by these substances [29]. Considering emulsification with the solvent evaporation method, the identified EE% of 38.0–86.2% was considered suitable for further studies. 

A previous study on clobetasol propionate-loaded lecithin-chitosan NPs demonstrated the encapsulation and accumulation in vitro was dependent on the positive charge of polymer [30]. Further studies using the same method have shown less satisfactory results of drug incorporation. However, this can be solved by changing some parameters of the nanoencapsulation method. Topotecan and thymoquinone were encapsulated by using a modified double-emulsion solvent evaporation method, which resulted in an EE% of 42.3%–62.6% [31]. The drug-loading efficiency of ciprofloxacin in the lipid-polymer hybrid NPs, produced by using the emulsification-solvent evaporation method, was between 53% and 72% [32]. In addition, all produced formulations exhibited a narrower particle size distribution and spherical shape (Figure 2). The zeta potential of approximately +25 mV suggested a stable colloidal dispersion [33]. AFM images and DLS measurements demonstrate the excellent performance of optimized formulations, which is not a trivial fact [34,35,36].

The FTIR data (Figure 3) corroborated with the structural differences identified in Table 1. Perturbation on the surface of NPs generally affects drug/copolymer interactions, causing changes in the spectra [37]. The enhancement of marked bands in drug-loaded and SA- and Chol- functionalized drug-loaded NPs further supported this. This occurred in the ester stretching from the copolymer (1730 cm^−1^), as well as in stretching bands in the region of 1365–1246 cm^−1^, due to the stretching of the nitro group and the angular C-H deformation, respectively. These results were suggestive of not only the perturbation on the NP surface, but also that the drug on the surface of the particles was induced by SA and Chol functionalization. These different drug interactions with compounds in NPs can induce different drug release rates that NPs can supply [38,39].

An exploration of the possible instability phenomena in the colloidal dispersion is presented in Figure 3. Phenomena such as flocculation, creaming, sedimentation, or coalescence induced by drug-loading or functionalization not were detected in this experiment [40,41]. There were no significant variations in either particle size or zeta potential over an interval of 6 weeks (*p* > 0.05), which corroborated with the successful preparation of NPs able to improve the drug efficacy [42,43]. Schamp and Jesser [44] observed a planar interface separating two distinct phases in 10–40 nm diameter Bi-Sn NPs (smaller NPs were of a single phase). Howe et al. [45] also noticed that an Ag-Cu NPs phase separated into two spherical layers in a few days, although the NPs analyzed were much larger (100 nm in diameter), which demonstrated the importance of evaluating the stability of colloidal nanocarrier systems.

As shown in Figure 4, SA or Chol functionalization enhanced drug release from NPs. These data corroborated with the FTIR data, which suggested that the competition of SA or Chol with the drug in the core of particles induced the transport of BNZ to the NP surface. The release data for the functionalized NPs were better fitted using the Bhaskar mathematical model, suggesting that drug release was controlled by diffusion [45]. A higher drug release rate constant (*k*) was observed for NP BNZ Chol than NP BNZ SA, which showed that the different functionalization induced different particle-cell interactions and biological activities [46]. A previous study with chitosan NPs functionalized with gallic acid showed similar results, evidencing that functionalization with appropriate substances induced relevant changes in the drug-release process [47].

The drug-release profile from non-functionalized NP formulations was better fitted by using the parabolic diffusion model. This suggested that the drug release process was controlled by diffusion from within the particle or the surface of NPs [48]. Previous studies with self-assembled cross-linked chitosan NPs containing scorpion venom proteins for immunotherapy also demonstrated a similar diffusion profile of proteins [49].

Unlike from NPs, the release of free BNZ was very fast, as shown in Figure 4A. The slow and prolonged release of BNZ from NPs was consistent with their evaluated physicochemical properties. The ability of colloids to retain the drug trapped in the polymer matrix improved the biological activity of the BNZ, as the drug is not released before arriving at the specific site of action [50,51].

The cell viability results shown in Figure 5 demonstrate that NPs without BNZ were less cytotoxic and, possibly, did not affect the metabolism and viability of normal cells. This suggests that drug-free NPs did not exhibit severe cytotoxicity at the tested concentration range [35]. The difference in the combined action of transporters and metabolic enzymes between normal and carcinogenic cell lines may also be a factors to be considered [52,53,54,55].

The NPs containing BNZ, including the functionalized NPs, displayed higher efficacy against cancer cells. This occurred owing to greater uptake into cancer cells than into HEK-293 cells. In tumoral cell lines, such as HT-29 and HeLa, the increase in the effectiveness of the drug was more evident for the NPs functionalized with SA and Chol (cell viability < 50%). The main advantage of NPs is their ability to overcome the biological barriers in the cancer, which improves drug efficacy [56]. The enhanced permeability and retention (EPR) effect of NPs is conferred by their biodistribution and passive targeting to the affected tissue, where the uptake of particles occurs by endocytosis. Certain molecules anchored on the NPs can improve endocytosis by interaction with specific co-transporter systems [57,58]. SA and Chol, used to enhance the surface of the particles, are good candidates for this as they are essential for cell proliferation [59,60]. 

In addition, cationic NPs can enhace adsorptive-mediated endocytosis owing to their favorable electrostatic interaction with anionic phospholipids [57,58]. However, this favorable interaction should be modulated, because it can induce defects in the lipid bilayer and cell disruption [61]. In the in vivo experiments, the exacerbated interaction of cationic NPs with the cell membrane or their intensive uptake can induce oxidative stress that mainly affects the hepatic sinusoid with Kupffer cells and the renal glomerular basement membrane, resulting in hepatic and renal toxicities [62]. The generation of ROS was also reported in the lungs and associated with pulmonary inflation [63]. Some studies have correlated the cytotoxity of cationic polymers, such as poly(amidoamine) (PAMAM) dendrimers, with the number of primary amino groups [64]. However, it is possible to modulate the cytotoxicity, dependent on the cationic charge, by shielding cationic groups with specific molecules during the functionalization of particles [65,66]. 

Thus, the cell viability results demonstrated that Eudragit NPs did not induce considerable cytotoxicity at the concentation range tested. However, owing to its polycationic character, the EUD Eudragit is not considered a good candidate for use in NPs in vivo studies. In previous studies, the in vivo efficacy of Eudragit NPs was demonstrated after oral administration, but safe use by the parenteral route has not yet been elucidated. Thus, the conceptaul base discussed in this study allows us to explore the potential use of cationic NPs, decorated with SA or Chol, to improve the efficacy of BNZ. Further in vivo studies should be performed using polymers approved for parenteral use, such as aliphatic polyesters with a positive charge modulated by a specific agent. 

## 4. Materials and Methods

### 4.1. Materials

Benznidazole (BNZ) (Roche^®^, São Paulo, Brazil) was donated by LAFEPE^®®^—Pharmaceutical Laboratory of Pernambuco (Recife, Brazil); poly vinyl alcohol (PVA) with viscosimetric molecular mass of 4.7 × 10^4^ g/mol (Vetec^®^, São Paulo, Brazil); copolymer of Polymethylmethacrylate (PMMA), Eudragit^®®^ E PO (EUD) was purchased from Pharma Polymers (Röhm GmbH & Co. KG, Darmstadt, Germany); SA (N-Acetylneuraminic acid, Neu5Ac, >98%, CarboSynth, Compton, Berkshire, England) and Chol (92.5%, Vetec^®®^, Sigma-Aldrich, Saint Louis, MO, EUA). The organic solvents ethanol (EtOH) (ɛ of 24.6), acetone (ACE) (ɛ of 20.6), and dichloromethane (DCM) (dielectric Constant (ɛ) of 9.1) were purchased from Labsynth^®®^ (São Paulo, Brazil). Both 3-(4,5-Dimethylthiazol-2-yl)-2,5-Diphenyltetrazolium Bromide (MTT) and dimethyl sulfoxide (DMSO) were purchased from Sigma Aldrich (Saint Louis, CA, USA). The purified water (1.3 μS) was prepared by equipment model OS50 LX (Gehaka, SP, Brazil) from reverse osmosis purification. All other chemicals and reagents were of analytical grade.

### 4.2. Preparation of Surface-Modified Chol/SA NPs

The cationic NPs were prepared using a previously established emulsification solvent evaporation method [67,68,69]. The PMMA 0.5% *w*/*v* (5 mg/ mL) solution of organic phase (6 mL) composed by DCM:EtOH at 50:50 *v*/*v* was dripped in the PVA 0.25% *w*/*v* (2.5 mg/mL) aqueous solution (14 mL), at 1.0 mL/min under magnetic stirring at 720 rpm. The emulsification followed in ultra turrax equipment (IKA Labortechnik, Staufen, Germany) at 20,000 rpm for 18 min. The solvent evaporation occurred at 25 °C under magnetic stirring at 720 rpm overnight. Samples were stored in hermetically sealed glass flasks and stored at 5 °C. The BNZ-loaded NPs (NP BNZ) were prepared using drugs dissolved in the organic phase to obtain a drug/copolymer ratio of 1:10 *w*/*w* (0.0125 mg/ 25 mL) [2]. The functionalized NPs Chol (NP BNZ Chol) were prepared using Chol/copolymer ratios of 1:40, 1:20, and 1:10 *w*/*w*, which corresponded respectively to 0.125 mg/ mL, 0.25 mg/mL, and 0.5 mg/ mL of Chol at the organic phase (6 mL). The SA (NP BNZ SA) were prepared using the same SA/copolyner ratio of 1:40, 1:20, and 1:10 *w*/*w*, which correponded respectively to 0.05 mg/mL, 0.11 mg/mL, and 0.22 mg/mL of SA dissolved at the aqueus phase (14 mL). All experiments were performed in triplicate with their physicochemical parameters measured and expressed as mean ± standard deviation (SD).

### 4.3. Particle Size and Zeta Potential Measurements

The mean particle size and polydispersity index (PdI) were evaluated using dynamic light scattering (DLS) at 659 nm, with a detection angle of 90°, on a particle size analyzer (Brookhaven Instruments, Long Island, NY, USA), at 25 °C. The zeta potential measurements were performed on the same equipment, applying a field strength applied around 5.9 V cm^−1^ and using Zeta Plus^®^ software Particle Sizing version 3.95. Samples were diluted 1:50 (*v*/*v*) with purified water using ten measurements in triplicate. Data were expressed as mean ± standard deviation (SD).

### 4.4. Attenuated Total Reflectance Fourier Transforms Infrared Spectroscopy (ATR–FTIR)

Colloidal NPs were concentrated by using a vacuum concentrator (Labconco Centrivap, Kansas City, MO, USA) for about 7 h to dry the samples. The spectra were recorded at 20 scans, with a resolution of 4 cm^−1^ between 4000 and 500 cm^−1^ for pure compounds (BNZ, PMMA, SA, and Chol) and NPs (NP, NP BNZ, NP BNZ SA, and NP BNZ Chol) in a FTIR-ATR spectrophotometer (SHIMADZU IR Prestige 21, Tokyo, Japan).

### 4.5. Atomic Force Microscopy (AFM)

The shape and surface of NPs were observed by using AFM images. The drug-free, drug loaded NPs, and functionalized drug loaded NPs were freshly diluted with purified water in ratio 1:100 (*v*/*v*) and dropped in a cover slip, dried in desiccator for 24 h, and then analyzed in an AFM, SPM-9700, Shimadzu (Tokyo, Japan), with a non-contact cantilever and 1Hz scanning at room temperature.

### 4.6. Drug-Loading Efficiency

The samples were centrifuged for 60 min, at 16.0 × g, and at 4 °C using the ultracentrifuge filter (Sartorius^®^, Vivaspin 2, Ultra-15 MWCO 100 kDa, Goettingen, Germany). The drug in the supernatant was analyzed in the length of maximum absorption of drug, which is at 324 nm, using previously validated UV-Vis spectrophotometry (Termo Fisher scientific, Whalthan, Massachusets, USA). All analyses were expressed as mean ± standard deviation (SD) and performed in triplicate. The encapsulation efficiency (EE) and the drug loading (DL) was calculated by using Equations (1) and (2), as follows:

EE (%) = (total drug mass − drug mass in supernatant)/total drug mass × 100,
(1)

DL (%) = (drug mass in NPs/mass of NPs) × 100.
(2)

### 4.7. Physicochemical Stability Study

The free, drug-loaded, and functionalized NPs were stored for six weeks in hermetically sealed vials and conditioned at 5 °C. Each day the samples were collected and evaluated for size and zeta potential. Measurements were performed at 25 °C using the parameters described in Section 2.4. All data were expressed as mean ± standard deviation (SD) and analyzed in triplicate.

### 4.8. In Vitro Drug Release

The in vitro BNZ release study was done with thermostatized vertical Franz diffusion cells (Crown Scientific, Sommerville, USA) at 37 ± 0.5 °C. In the donor compartment was inserted 3.0 mL of the different NPs containing the drug, as well as the BNZ solution. The compartment was hermetically sealed and separated from the recipient compartment by a 0.45 μm synthetic cellulose acetate filter (previously hydrated in a phosphate buffer for 24 h). The receiver compartment was filled with phosphate buffer solution adjusted to pH 7.4, which remained under magnetic stirring at 360 rpm for the entire experiment. At determined intervals, aliquots of 2.0 mL were removed and analyzed by previously validated 324 nm UV spectrophotometry. At each sample collection, the same volume of buffer replaced the medium to maintain the sink conditions. All analyses were performed in triplicate and data were expressed as mean ± standard deviation (SD).

### 4.9. In Vitro Drug Release Kinetics

For comparison of drug release rate and elucidating the possible mechanism of drug release frUSAom NPs, the experimental data of in vitro drug release were fitted using the four following linear kinetic models [70,71,72]. All calculations were performed in triplicate and data is expressed as mean ± standard deviation (SD).

(i) The first-order that describes BNZ release rate dependent on drug amount in the cationic NPs, expressed as

ln(M_t_/M∞) − kt. (3)

(ii) The Bhaskar model that describes whether the drug diffusion through the particle is the rate limiting step, expressed as

lg(M_t_/M∞)= −Bt^0.65^. (4)

(iii) The modified Freundlich model that describes the drug release from a flat surface with heterogeneous sites based on a diffusion-controlled process, expressed as

(M∞ − M_t_)/M∞ = kt^b^. (5)

(iv) The parabolic diffusion model describes that the drug release process is controlled by a diffusion from intra-particle or surface of NPs, expressed as

(1 − M_t_/M∞)/t = kt^−0.5^ + a. (6)

In the previously described equations, Mt/M∞, t, k are the fraction released drug, release time, the drug release rate constant, respectively. The B is drug release rate constant of Bhaskar, while a and b are constants whose chemical significance is not clearly solved.

### 4.10. In Vitro Anticancer Efficacy

#### 4.10.1. Cell Culture

The normal renal cell line (HEK-293), human hepatocyte carcinoma (HepG2), human colorectal cancer (HT-29), and human cervical carcinoma (HeLa) lines were obtained from the Federal University of Rio de Janeiro (RJCB Collection, Rio de Janeiro, Brazil). All cells were maintained in Dulbecco’s modified Eagle’s medium (DMEM) supplemented with 10% (*v*/*v*) fetal bovine serum (CULTILAB LTD, São Paulo, Brazil), conditioned in a humid atmosphere containing 5% CO_2_ at 37 °C. Cell maintenance was performed every 3 days or according to the needs of the strains. At approximately 80% confluency, trypsin/EDTA cells (Gibco, São Paulo, Brazil) were counted with a Neubauer chamber, stained with trypan blue, and plated on 96 wells.

#### 4.10.2. Cytotoxity Assay

The viability of cells against the control (DMEM supplemented with 10% (*v*/*v*) fetal bovine serum), (Benznidazole solution (BNZ), free BNZ loaded NPs, BNZ-loaded NPs (NP BNZ), and BNZ-loaded functionalized NPs (NP BNZ SA and NP BNZ Chol) were evaluated through an in vitro cytotoxicity assay. For this, we used the quantitative method of MTT (3-methyl-[4-5-dimethylthiazol-2-yl]-2,5-diphenyltetrazolium bromide). Viable cells retain the capacity to reduce the MTT at formazan crystals due the succinate dehydrogenase enzyme activity found in the mitochondria [73]. All cell lines were plated in 96-well plates at a density of 5 × 10^3^ cells/well. After 24 h, the excess cells were removed and the study samples were added, which were pre-filtered using a 0.45 μm sterile Millipore filter (São Paulo, Brazil). BNZ was diluted in 1% DMSO, yielding a stock solution to be diluted in the following concentrations of BNZ: 62.4 μg/mL, 31.2 μg/mL, and 15.6 μg/mL. The same concentrations were used for NPs containing BNZ, as well as for functionalized NPs. The sample the incubation period was 24 and 48 h. The supernatant containing the samples was removed and 100 ul of MTT containing medium (final concentration 5 mg/ml) were added to each well. The plate was placed in a 37% CO_2_ oven for 4 h. After this time the MTT was removed and 100 μL of ethanol was added to each well. The plate was protected from light and placed under stirring for 15 min, then the absorbance was measured spectrophotometrically in microtiter plate reader at 570 nm (Epoch-BioTek, Winooski, USA [74].

### 4.11. Statistical Analysis

The Student’s t-test was used for paired comparisons of the analytical data. Univariate analysis of variance (ANOVA) was applied for multiple comparisons, followed by the Dunnett or post hoc Tukey test. Cell viability assay data were compared using one-way analysis of variance followed by Bonferroni’s test. Values of *p* < 0.05 were considered statistically significant and were expressed as mean ± standard deviation (SD).

## 5. Conclusions

In this study, we explored two natural compounds SA and Chol, as possible candidates for the functionalization of cationic NPs. The preparation parameters and physicochemical properties of stable sub-200 nm NPs were controlled and monitored. All formulations were biocompatible in normal kidney cells (HEK 293) and improved BNZ efficacy, compared to the free drug. The functionalization of BNZ-NPs improved the anticancer effect in human carcinoma cervical cancer (HeLa) and human colorectal cancer (HT-29) cells, with Chol functionalization offering better performance. The results discussed in this approach provide practical gains to develop promising BNZ-loaded NPs with anticancer effects. 

## Figures and Tables

**Table 1:** Physicochemical properties of drug-free NPs, drug-loaded NPs (NP BNZ), and functionalized NP BNZ formulations.

Samples	Drug Loading (%)	Size (nm) ± SD	PdI ± SD	Zeta Potential (mV) ± SD	EE (%) ± SD
NP	ND	148.0 ± 2.4	0.189 ± 2.1	+25.2 ± 0.3	ND
NP BNZ	4.0	154.2 ± 1.0	0.254 ± 1.0	+22.1 ± 0.1	86.2 ± 2.3
NP BNZSA	3.7	176.9 ± 1.2	0.239 ± 1.6	+21.7 ± 0.1	47.5 ± 1.1
NP BNZ Chol	3.7	186.8 ± 2.5	0.238 ± 2.5	+20.0 ± 0.4	38.6 ± 1.7

Note: ND = no drug; SD = standard deviation; PdI = polydispersity index; EE% = encapsulation efficiency.

**Table 2:** Fitting parameters for different kinetic models applied to the in vitro BNZ release data from cationic NPs.

Formulations	Kinetic Models [k value (r^2^ ± SD)]			
	First-Order	Bhaskar	Freundlich	Parabolic Diffusion
NP BNZ	−0.00008 h^−1^ (0.55 ± 0.04)	−0.0004 h^0.65^ (0.63 ± 0.02)	0.0578 h (0.85 ± 0.02)	−0.026 h^−0.5^ (0.99 ±0.03)
NP BNZ SA	−0.0021 h^−1^ (0.86 ± 0.03)	−0.0095 h^0.65^ (0.94 ± 0.02)	−0.4246 h (0.86 ± 0.02)	0.0016 h^−0.5^ (0.16 ±0.03)
NP BNZ CHOL	−0.0023 h^−1^ (0.83 ± 0.03)	−0.0114 h^0.65^ (0.93 ± 0.02)	−0.5117 h (0.85 ± 0.02)	−0.0064 h^−0.5^ (0.41 ± 0.03)

Notes: Drug release rate constant (k), (r²) correlation coefficient. The data are expressed as the mean of three determinations.

## References

[1] Yokoyama C., Sueyoshi Y., Ema M., Mori Y. (2017). Induction of oxidative stress by anticancer drugs in the presence and absence of cells. Oncol. Lett..

[2] Santos F.M., Mazzeti A.L., Caldas S., Gonçalves K.R., Lima W.G., Torres R.M., Bahia M.T. (2016). Chagas cardiomyopathy: The potential effect of benznidazole treatment on diastolic dysfunction and cardiac damage in dogs chronically infected with Trypanosoma cruzi. Acta Trop..

[3] Hall B.S., Wilkinson S.R. (2012). Activation of benznidazole by trypanosomal type I nitroreductases results in glyoxal formation. Antimicrob. Agents Chemother..

[4] Manarin R., Pascutti M.F., Ruffino J.P., De Las Heras B., Boscá L., Bottasso O., Revelli S., Serra E. (2010). Benznidazole blocks NF-kB activation but not AP-1 through inhibition of IKK. Mol. Immunol..

[5] Dos Santos-Silva A.M., de Caland L.B., de S L Oliveira A.L.C., de Araújo-Júnior R.F., Fernandes-Pedrosa M.F., Cornélio A.M., da Silva-Júnior A.A. (2017). Designing structural features of novel benznidazole-loaded cationic nanoparticles for inducing slow drug release and improvement of biological efficacy. Mater. Sci. Eng. C.

[6] Moghimi S.M., Hunter A.C., Andresen T.L. (2012). Factors Controlling Nanoparticle Pharmacokinetics: An Integrated Analysis and Perspective. Annu. Rev. Pharm. Toxicol..

[7] Bamrungsap S., Zhao Z., Chen T., Wang L., Li C., Fu T., Tan W. (2012). Nanotechnology in therapeutics: A focus on nanoparticles as a drug delivery system. Nanomedicine.

[8] Guzmán M.L., Manzo R.H., Olivera M.E. (2012). Eudragit E100 as a drug carrier: The remarkable affinity of phosphate ester for dimethylamine. Mol. Pharm..

[9] Jana U., Mohanty A.K., Pal S.L.A.L., Manna P.K., Mohanta G.P. (2014). Preparation and in vitro characterization of Felodipine loaded Eudragit^®^ RS100 nanoparticles. Innovare Acad. Sci..

[10] Li S.D., Huang L. (2009). Nanoparticles evading the reticuloendothelial system: Role of the supported bilayer. Biochim. Biophys. Acta.

[11] Blanco E., Shen H., Ferrari M. (2015). Principles of nanoparticle design for overcoming biological barriers to drug delivery. Nat. Biotechnol..

[12] Sajeesh S., Lee T.Y., Kim J.K., Son D.S., Hong S.W., Kim S., Yun W.S., Kim S., Chang C., Li C. (2014). Efficient intracellular delivery and multiple-target gene silencing triggered by tripodal RNA based nanoparticles: A promising approach in liver-specific RNAi delivery. J. Control. Release.

[13] Wasungu L., Hoekstra D. (2006). Cationic lipids, lipoplexes and intracellular delivery of genes. J. Control. Release.

[14] Chou L.Y., Ming K., Chan W.C. (2011). Strategies for the intracellular delivery of nanoparticles. Chem Soc. Rev..

[15] Fröhlich E. (2012). The role of surface charge in cellular uptake and cytotoxicity of medical nanoparticles. Int. J. Nanomed..

[16] Arvizo R.R., Miranda O.R., Moyano D.F., Walden C.A., Giri K., Bhattacharya R., Robertson J.D., Rotello V.M., Reid J.M., Mukherjeeet P. (2011). Modulating pharmacokinetics, tumor uptake and biodistribution by engineered nanoparticles. PLoS ONE.

[17] Mecke A., Majoros I.J., Patri A.K., Baker J.R., Holl M.M., Orr B.G. (2005). Lipid bilayer disruption by polycationic polymers: The roles of size and chemical functional group. Langmuir.

[18] Albanese A., Tang P.S., Chan W.C.W. (2012). The effect of nanoparticle size, shape, and surface chemistry on biological systems. Annu. Rev. Biomed. Eng..

[19] Luxenhofer R. (2015). Polymers and nanomedicine: Considerations on variability and reproducibility when combining complex systems. Nanomedicine (Lond.).

[20] Sapra M., Pawar A.A., Venkataraman C. (2016). A single-step aerosol process for in-situ surface modification of nanoparticles: Preparation of stable aqueous nanoparticle suspensions. J. Colloid Interface Sci..

[21] Kobayashi K., Wei J., Iida R., Ijiro K., Niikura K. (2014). Surface engineering of nanoparticles for therapeutic applications. Polym. J..

[22] Bondioli L., Costantino L., Ballestrazzi A., Lucchesi D., Boraschi D., Pellati F., Benvenuti S., Tosi G., Vandelli M. (2010). PLGA nanoparticles surface decorated with the sialic acid, *N*-acetylneuraminic acid. Biomaterials.

[23] Schofield C.L., Marín M.J., Rejzek M., Crocker P.R., Field R., Russell D. (2016). Detection of mSiglec-E, in solution and expressed on the surface of Chinese hamster ovary cells, using sialic acid functionalised gold nanoparticles. Analyst.

[24] Miller B.R., Roitberg A.E. (2013). Trypanosoma cruzi trans-sialidase as a drug target against Chagas disease (*American trypanosomiasis*). Future Med. Chem..

[25] Lee J.J., Lee S.Y., Park J.H., Kim D.D., Cho H.J. (2016). Cholesterol-modified poly(lactide-*co*-glycolide) nanoparticles for tumor-targeted drug delivery. Int. J. Pharm..

[26] Belletti D., Grabrucker M., Pederzoli F., Menrath I., Cappello V., Vandelli M., Forni F., Tosi G., Ruozi B. (2016). Exploiting the Versatility of Cholesterol in Nanoparticles Formulation. Int. J. Pharm..

[27] Xie J., Pan X., Wang M., Ma J., Fei Y., Wang P.N., Mi L. (2016). The role of surface modification for TiO_2_ nanoparticles in cancer cells. Colloids Surf. B Biointerfaces.

[28] Mobarak D.H., Salah S., Elkheshen S. (2014). Formulation of ciprofloxacin hydrochloride loaded biodegradable nanoparticles: Optimization of technique and process variables. Pharm. Dev. Technol..

[29] Senyi T., Sonvico F., Rossi A., Santi P., Colombo P., Nicoli S., Özer Ö. (2017). In Vivo Assessment of Clobetasol Propionate-Loaded Lecithin-Chitosan Nanoparticles for Skin Delivery. Int. J. Mol. Sci..

[30] Verma D., Thakur P.S., Padhi S., Khuroo T., Talegaonkar S., Iqbal Z. (2017). Design expert assisted nanoformulation design for co-delivery of topotecan and thymoquinone: Optimization, in vitro characterization and stability assessment. J. Mol. Liq..

[31] Dave V., Yadav R.B., Kushwaha K., Yadav S., Sharma S., Agrawal U. (2017). Lipid-polymer hybrid nanoparticles: Development & statistical optimization of norfloxacin for topical drug delivery system. Bioact. Mater..

[32] Murakami H., Kobayashi M., Takeuchi H., Kawashima Y. (1999). Preparation of poly(dl-lactide-*co*-glycolide) nanoparticles by modified spontaneous emulsification solvent diffusion method. Int. J. Pharm..

[33] Gocalinska A., Manganaro M., Dimastrodonato V., Pelucchi E. (2015). Evaluation of defect density by top-view large scale AFM on metamorphic structures grown by MOVPE. Appl. Surf. Sci..

[34] Hoo C.M., Starostin N., West P., Mecartney M.L. (2008). A comparison of atomic force microscopy (AFM) and dynamic light scattering (DLS) methods to characterize nanoparticle size distributions. J. Nanoparticle Res..

[35] Lim J., Yeap S.P., Che H.X., Low S.C. (2013). Characterization of magnetic nanoparticle by dynamic light scattering. Nanoscale Res. Lett..

[36] Hao S., Wang B., Wang Y., Zhu L., Wang B., Guo T. (2013). Preparation of Eudragit L 100-55 enteric nanoparticles by a novel emulsion diffusion method. Colloids Surf. B Biointerfaces.

[37] Jana U., Mohanty A.K., Manna P.K., Mohanta G.P. (2014). Preparation and characterization of nebivolol nanoparticles using Eudragit^®^ RS100. Colloids Surf. B Biointerfaces.

[38] Kang T., Jang I., Oh S.-G. (2016). Surface modification of silica nanoparticles using phenyl trimethoxy silane and their dispersion stability in *N*-methyl-2-pyrrolidone. Colloids Surf. A Phys. Eng. Asp..

[39] Lemarchand C., Couvreur P., Vauthier C., Costantini D., Gref R. (2003). Study of emulsion stabilization by graft copolymers using the optical analyzer Turbiscan. Proc. Int. J. Pharmaceut..

[40] Terayama H., Hirota K., Yoshimura T., Esumi K. (2003). Effect of dilution on aqueous dispersion of drug particles. Colloids Surf. B Biointerfaces.

[41] Perez A., Santiago L.G. (2017). Food Hydrocolloids Formation and colloidal stability of ovalbumin-retinol nanocomplexes. Food Hydrocoll..

[42] Akbarzadeh H., Abbaspour M., Salemi S., Hasani M. (2017). Coalescence process of gold/silver core-shell nanoparticles located on carbon nanotube and graphene surfaces. J. Mol. Liq..

[43] Schamp C.T., Jesser W.A. (2006). Two-phase equilibrium in individual nanoparticles of Bi-Sn. Metall. Mater. Trans. A Phys. Metall. Mater. Sci..

[44] Howe J.M., Mebed A.M., Chatterjee K., Li P., Murayama M., Johnson W.C. (2003). Effect of phase fraction on the tri-junction in two-phase nanoparticle systems. Acta Mater..

[45] Qi F., Zhang X., Li S. (2013). A novel method to get methotrexatum/layered double hydroxides intercalation compounds and their release properties. J. Phys. Chem. Solids.

[46] Lamarra J., Giannuzzi L., Rivero S., Pinotti A. (2017). Assembly of chitosan support matrix with gallic acid-functionalized nanoparticles. Mater. Sci. Eng. C.

[47] Calvez V., Corrias L. (2008). The parabolic-parabolic Keller-Segel model in R2. Commun. Math. Sci..

[48] Rocha Soares K.S., Oliveira A.R., Daniele-Silva A., Glaucia-Silva F., Caroni A.L.P., Fernandes-Pedrosa M.F., da Silva-Júnior A.A. (2017). Self-assembled scorpion venom proteins cross-linked chitosan nanoparticles for use in the immunotherapy. J. Mol. Liq..

[49] Seju U., Kumar A., Sawant K.K. (2011). Development and evaluation of olanzapine-loaded PLGA nanoparticles for nose-to-brain delivery: In vitro and in vivo studies. Acta Biomater..

[50] Clementino A., Batger M., Garrastazu G., Pozzoli M., Del Favero E., Rondelli V., Gutfilen B., Barboza T., Sukkar M.B., Souza S.A.L. (2016). The nasal delivery of nanoencapsulated statins—An approach for brain delivery. Int. J. Nanomed..

[51] Oostendorp R.L., Beijnen J.H., Schellens J.H.M. (2009). The biological and clinical role of drug transporters at the intestinal barrier. Cancer Treat. Rev..

[52] Zhang X., Dong Y., Zeng X., Liang X., Li X., Tao W., Chen H., Jiang Y., Mei L., Feng S.S. (2014). The effect of autophagy inhibitors on drug delivery using biodegradable polymer nanoparticles in cancer treatment. Biomaterials.

[53] Zhang H., Guo H., Jin S., Wang P., Du Z., Ren F. (2019). Novel Targeted Anti-Tumor Nanoparticles Developed from Folic Acid-Modified 2-Deoxyglucose. Int. J. Mol. Sci..

[54] Khmara I., Koneracka M., Kubovcikova M., Zavisova V., Antal I., Csach K. (2016). Preparation of poly-l-lysine functionalized magnetic nanoparticles and their in fl uence on viability of cancer cells. J. Magn. Magn. Mater..

[55] Cheng Y., Morshed R.A., Auffinger B., Tobias A.L., Lesniak M.S. (2014). Multifunctional nanoparticlesfor brain tumor imaging and therapy. Adv. Drug Deliv. Rev..

[56] Bicker J., Alves G., Fortuna A., Falcão A. (2014). Blood-brain barrier models and their relevancefor a successful development of CNS drug delivery systems: A review. Eur. J. Pharm. Biopharm..

[57] De Moraes Profirio D., Pessine F.B.T. (2018). Formulation of functionalized PLGA nanoparticles with folic acid conjugated chitosan for carboplatin encapsulation. Eur. Pol. J..

[58] Schauer R. (2004). Sialic acids: Fascinating sugars in higher animals and man. Proc. Zool..

[59] Eiichi B. (2000). Synthesis and function of sialic acid-conjugated cholesterols as ganglioside analogs: Their reconstitution to liposomes and interaction with rat lymphocytes. Proc. Jpn. Acad..

[60] Leroueil P.R., Berry S.A., Duthie K., Han G., Rotello V.M., McNerny D.Q., Baker J.R., Orr B.G., Banaszak Holl M.M. (2008). Wide varieties of cationic nanoparticles induce defects in supported lipid bilayers. Nano Lett..

[61] Bodeweina L., Schmelter F., Di Fiore S., Hollert H., Fischer R., Fenske M. (2016). Differences in toxicity of anionic and cationic PAMAM and PPI dendrimers in zebrafish embryos and cancer cell lines. Toxic. App. Pharm..

[62] Knudsen K.B., Northeved H., Kumar P., Permin A., Gjetting T., Andresen T.L., Larsen S., Wegener K.M., Lykkesfeldt J., Jantzen K. (2015). In vivo toxicity of cationic micelles and liposomes. Nanomedicine.

[63] Naha P.C., Davoren M., Lyng F.M., Byrne H.J. (2010). Reactive oxygen species (ROS) induced cytokine production and cytotoxicity of PAMAM dendrimers in J774A.1 cells. Toxicol. Appl. Pharm..

[64] Mura S., Hillaireau H., Nicolas J. (2011). Influence of surface charge on the potential toxicity of PLGA nanoparticles towards Calu-3 cells. Int. J. Nanomed..

[65] Luo X., Feng M., Pan S., Wen Y., Zhang W., Wu C. (2012). Charge shielding effects on gene delivery of polyethylenimine/DNA complexes: PEGylation and phospholipid coating. J. Mater. Sci. Mater. Med..

[66] Lemos-Senna E., Wouessidjewe D., Lesieur S., Duchêne D. (1998). Preparation of amphiphilic cyclodextrin nanospheres using the emulsification solvent evaporation method. Influence of the surfactant on preparation and hydrophobic drug loading. Int. J. Pharm..

[67] Pooja D., Tunki L., Kulhari H., Reddy B.B., Sistla R. (2016). Optimization of solid lipid nanoparticles prepared by a single emulsification-solvent evaporation method. Data Br..

[68] Ma W., Chen M., Kaushal S., McElroy M., Zhang Y., Ozkan C., Bouvet M., Kruse C., Grotjahn D., Ichim T. (2012). PLGA nanoparticle-mediated delivery of tumor antigenic peptides elicits effective immune responses. Int. J. Nanomed..

[69] Gu Z., Thomas A.C., Xu Z.P., Campbell J.H., Lu G.Q. (2008). In vitro sustained release of LMWH from MgAl-layered double hydroxide nanohybrids. Chem. Mater..

[70] Yang J.H., Han Y.S., Park M., Park T., Hwang S.J., Choy J.H. (2007). New inorganic-based drug delivery system of indole-3-acetic acid-layered metal hydroxide nanohybrids with controlled release rate. Chem. Mater..

[71] Bhaskar R., Murthy R.S.R., Miglani B.D., Viswanathan K. (1986). Novel method to evaluate diffusion controlled release of drug from resinate. Int. J. Pharm..

[72] Son S., Kim W.J. (2010). Biodegradable nanoparticles modified by branched polyethylenimine for plasmid DNA delivery. Biomaterials.

[73] Lima K.M.G., Junior R.F.A., Araujo A., Oliveira A.L.C.S.L., Gasparotto L.H.S. (2014). Environmentally compatible bioconjugated gold nanoparticles as efficient contrast agents for colorectal cancer cell imaging. Sens. Actuators B Chem..

[74] De Melo P.N., Barbosa E.G., De Caland L.B., Carpegianni H., Garnero C., Longhi M., De Freitas Fernades-Pedrosa M., Da Silva-Júnior A.A. (2013). Host-guest interactions between benznidazole and beta-cyclodextrin in multicomponent complex systems involving hydrophilic polymers and triethanolamine in aqueous solution. J. Mol. Liq..

